# Quantitative study of 3T MRI qDixon-WIP applied in pancreatic fat infiltration in patients with type 2 diabetes mellitus

**DOI:** 10.3389/fendo.2023.1140111

**Published:** 2023-02-17

**Authors:** Jixing Yi, Fengming Xu, Tao Li, Bumin Liang, Shu Li, Qing Feng, Liling Long

**Affiliations:** ^1^ Department of Radiology, The First Affiliated Hospital of Guangxi Medical University, Guangxi Zhuang Autonomous Region, Nanning, China; ^2^ Department of Radiology, Fourth Affiliated Hospital of Guangxi Medical University, Liuzhou Worker’s Hospital Guangxi Zhuang Autonomous Region, Liuzhou, China; ^3^ School of International Education, Guangxi Medical University, Guangxi Zhuang Autonomous Region, Nanning, China

**Keywords:** multi-echo Dixon, magnetic resonance imaging, pancreatic fat infiltration, type 2 diabetes, quantitative study

## Abstract

**Objective:**

To investigate the application value of 3T MRI qDixon-WIP technique in the quantitative measurement of pancreatic fat content in patients with type 2 diabetes mellitus (T2DM).

**Methods:**

The 3T MRI qDixon-WIP sequence was used to scan the livers and the pancreas of 47 T2DM patients (experimental group) and 48 healthy volunteers (control group). Pancreatic fat fraction (PFF), hepatic fat fraction (HFF), Body mass index (BMI) ratio of pancreatic volume to body surface area (PVI) were measured. Total cholesterol (TC), subcutaneous fat area (SA), triglyceride (TG), abdominal visceral fat area (VA), high density lipoprotein (HDL-c), fasting blood glucose (FPC) and low-density lipoprotein (LDL-c) were collected. The relationship between the experimental group and the control group and between PFF and other indicators was compared. The differences of PFF between the control group and different disease course subgroups were also explored.

**Results:**

There was no significant difference in BMI between the experimental group and the control group (*P*=0.231). PVI, SA, VA, PFF and HFF had statistical differences (*P*<0.05). In the experimental group, PFF was highly positively correlated with HFF (*r*=0.964, *P*<0.001), it was moderately positively correlated with TG and abdominal fat area (*r*=0.676, 0.591, *P*<0.001), and it was weakly positively correlated with subcutaneous fat area (*r*=0.321, *P*=0.033). And it had no correlation with FPC, PVI, HDL-c, TC and LDL-c (*P*>0.05). There were statistical differences in PFF between the control group and the patients with different course of T2DM (*P*<0.05). There was no significant difference in PFF between T2DM patients with a disease course ≤1 year and those with a disease course <5 years (*P*>0.05). There were significant differences in PFF between the groups with a disease course of 1-5 years and those with a disease course of more than 5 years (*P*<0.001).

**Conclusion:**

PVI of T2DM patients is lower than normal, but SA, VA, PFF, HFF are higher than normal. The degree of pancreatic fat accumulation in T2DM patients with long disease course was higher than that in patients with short disease course. The qDixon-WIP sequence can provide an important reference for clinical quantitative evaluation of fat content in T2DM patients.

## 1 Introduction

Type 2 diabetes mellitus(T2DM) is the most common type of diabetes mellitus (1, 2). Pancreatic fat infiltration may play an important role in the occurrence and development of T2DM (3, 4). The degree of lipid infiltration in the pancreas is closely related to abnormal lipid metabolism. With β-cell dysfunction and defective insulin secretion, lipid oxidation and lipolysis are inhibited, which leads to the increase of lipid deposition in the pancreas. The increased degree of pancreatic fat infiltration promotes the development of T2DM (5–7). Therefore, monitoring pancreatic fat content in T2DM patients may provide a certain reference for clinical evaluation of efficacy and disease progression.

Although pancreatic biopsy is the “golden standard” for the quantitative determination of pancreatic fat content, due to the fact that this method only provides small tissue samples, the final measured pancreatic fat content may vary with the different range and degree of pancreatic fat infiltration. Moreover, its invasiveness and poor patient compliance limit the regular detection of pancreatic fat content in T2DM patients. the pancreas is a retroperitoneal organ surrounded by abundant blood vessels and intestines, which makes puncture more difficult (8, 9).

In recent years, multi-echo dixon technology based on Magnetic resonance image (MRI), which is safe, non-invasive and has good tissue resolution, has been confirmed in various organs including the pancreas in terms of tissue fat quantification (10–13). The early two-point Dixon technique could only quantify the adipogenic variation below 50% (14), which was greatly affected by the non-uniformity of the main magnetic field and the attenuation effect of T1 and T2* (15). Three-point Dixon technique can collect one more in-phase echo signal on the basis of two-point method, which can correct T2* attenuation to a certain extent. However, the obtained organ fat fraction is susceptible to various confounding factors, and its accuracy and repeatability are not enough to be a reliable index of fat quantification (16). 6 Echo Dixon (qDixon) technology, compared with the earlier Dixon technology, effectively corrects the errors caused by the magnetic field inhomogeneity and T2* attenuation, making the quantitative results more accurate. The fat distribution map can not only directly measure the fat content quantitatively, but also fully reflect the fat distribution (17).

The purpose of this study was to investigate the value of 3T qDixon technique in the quantitative determination of pancreatic fat content in T2DM patients, and to provide reference for the early diagnosis, clinical treatment, disease progression and efficacy evaluation of pancreatic changes in T2DM patients by comparing the relationship between relevant indicators.

## 2 Materials and methods

### 2.1 Research objects

A total of 95 volunteers were recruited from April 1, 2019 to June 30, 2022, including 36 females (17 T2DM patients, 19 normal controls) and 59 males (30 T2DM patients, 29 normal controls). T2DM Patients ranged from 32 to 71 years old (51.32 ± 10.60). The normal control group ranged from 31 to 68 years old (51.28 ± 8.91). Inclusion criteria: (1) patients diagnosed with T2DM and healthy volunteers with similar age to T2DM patients ( ± 3 years old) and no related diseases. Exclusion criteria: (1) patients unable to participate in MRI examination due to contraindications or other reasons; (2) patients with liver and pancreatic tumors; (3) patients after splenectomy; (4) patients with abnormal metabolic function or metabolic diseases excluding T2DM; (5) patients with hepatitis virus or hepatitis B, and liver iron deposition; (6) patients with liver trauma or patients receiving a liver transplant; (7) patients with pancreatic inflammation and alcoholics; (8) Patients with a history of drug therapy for the the pancreas (Sulfonamides, azathioprine, glucocorticoids, thiazide diuretics) and liver (Platinum agents, antibiotics, alkylating agents, antipsychotics, anti-tuberculosis drugs, and anti-tumor drugs) within six months. This study was conducted in accordance with the principles of the Declaration of Helsinki and approved by the hospital Ethics Committee (NO.2022-E460-01).

### 2.2 Instruments and methods

MRI scans were performed on all subjects by the same operator with 10 years of extensive MRI scanning experience. Abdominal axial scan was performed at the end of breath using a 3.0T MRI scanner Siemens 3T MRI scanner (Prisma, Siemens Healthcare, Erlangen, Germany). qDdixon-WIP sequence scanning parameters: echo time (TE): 1.26, 2.60, 3.94, 5.28, 6.62, 7.96ms; repetition time (TR): 9.25ms; slice thickness: 3.5mm, matrix: 160×120; bandwidth: 1040Hz/Pixel; field of view: 380mm×313.5mm; scanning time: about 18s.

### 2.3 Image processing

**Figure 1:**
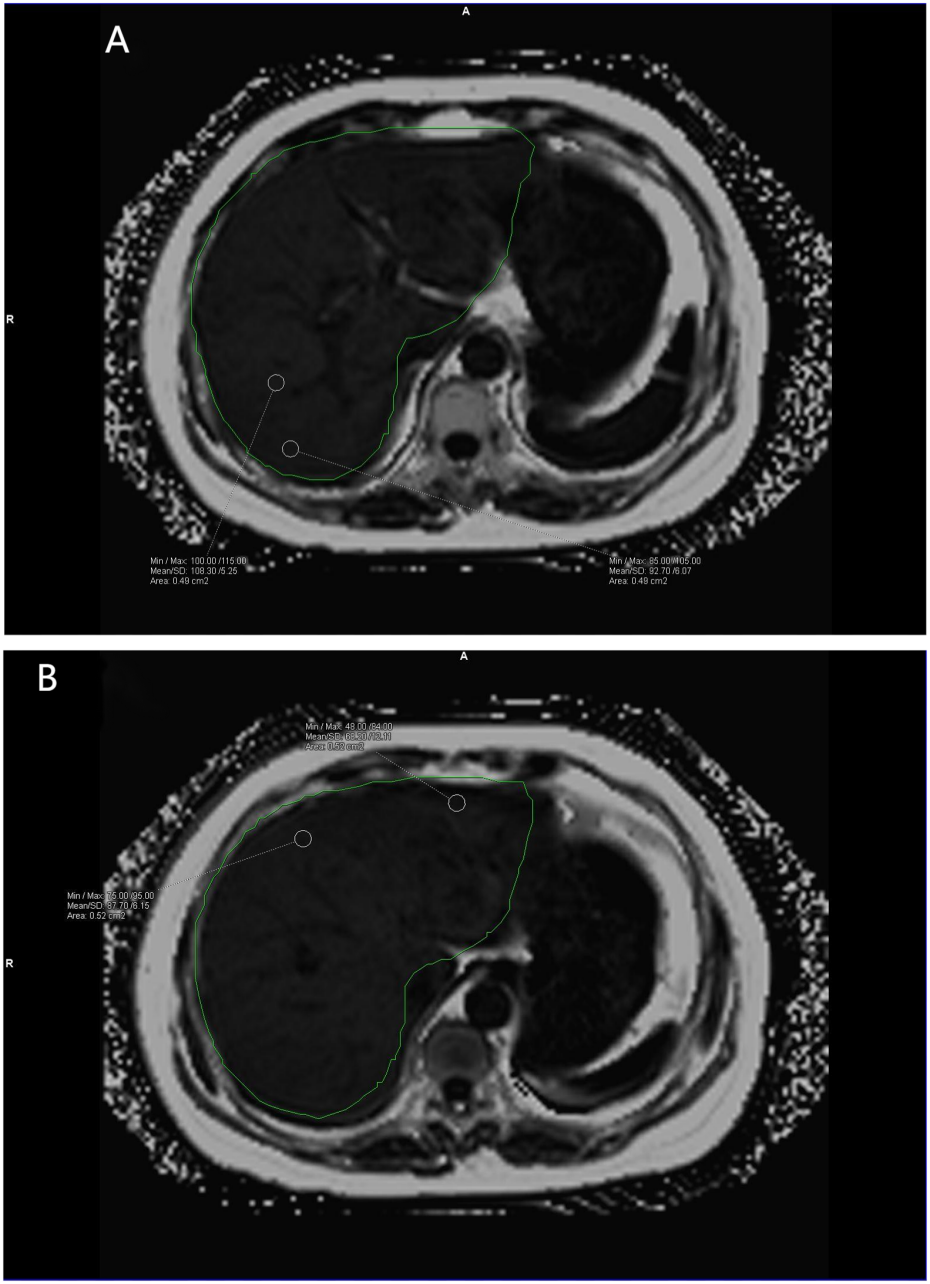
**(A, B)** show the liver fat fraction maps of volunteers. Mean fat fraction =10%× (108.30 + 92.70+68.20+87.70)/4 = 8.92 (two decimal places reserved). The green area is the liver region automatically delineated by the software.

**Figure 2:**
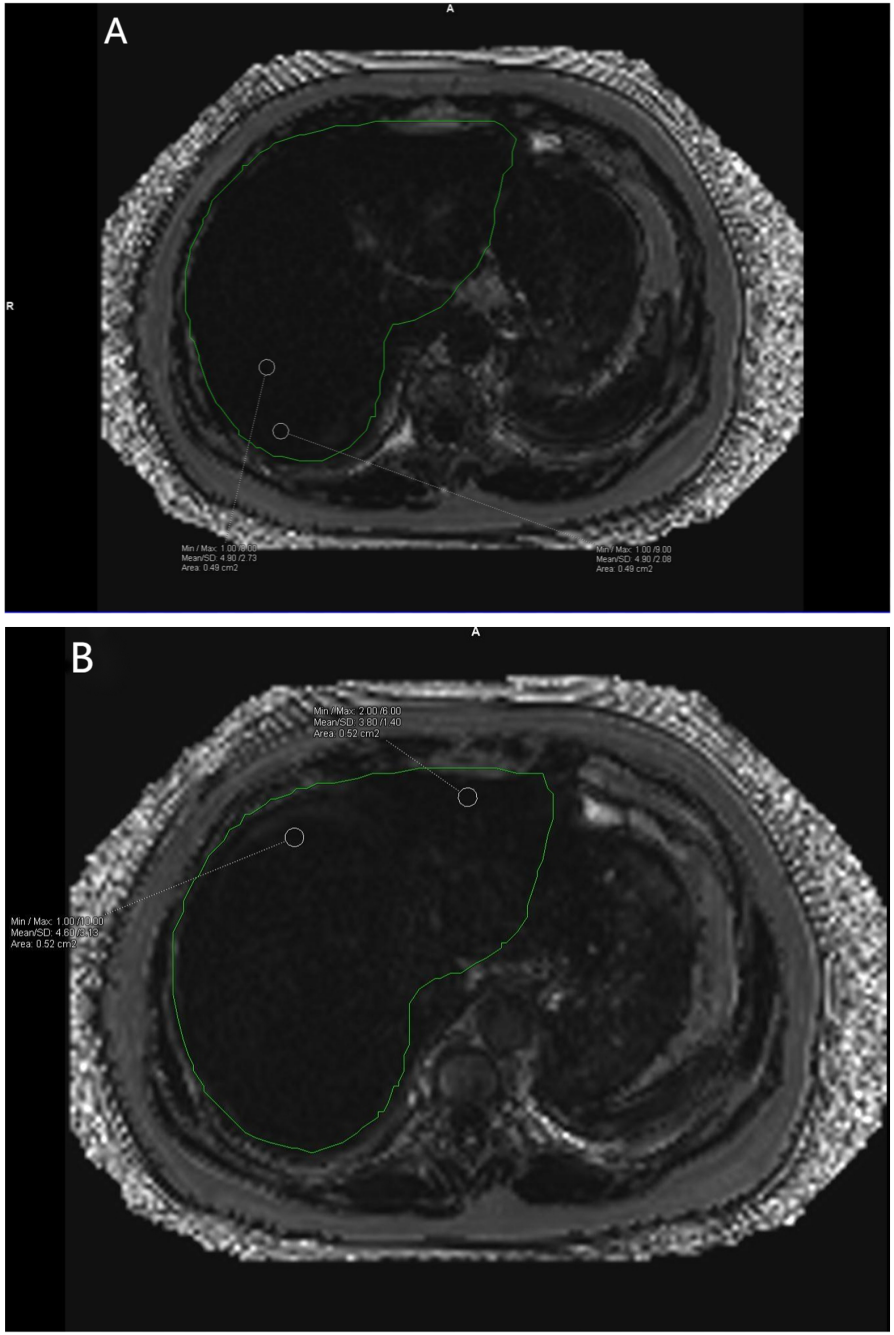
**(A, B)** show the corresponding Goodness of fit plots for liver fat fraction. A Goodness of fit average = (4.90% + 4.90% + 4.60% + 3.80%)/4 = 4.55%. The green area is the liver region automatically delineated by the software.

**Figure 3:**
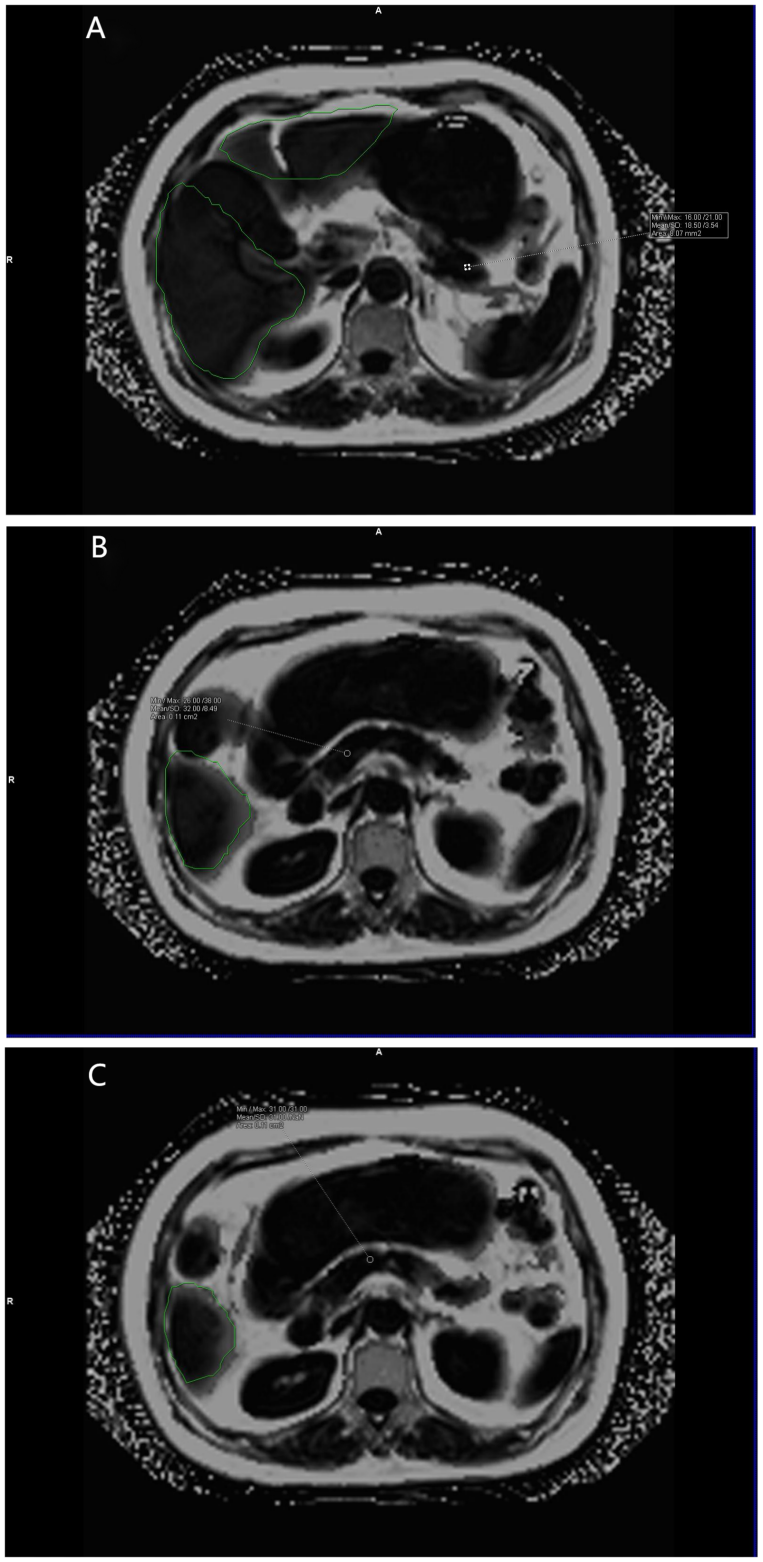
**(A–C)** show the pancreatic fat fraction maps of the volunteers. Mean pancreatic fat fraction =10%× (18.50 + 32.00+31.00)/3 = 2.72 (keep two decimal places). The green area is the liver region automatically delineated by the software.

**Figure 4:**
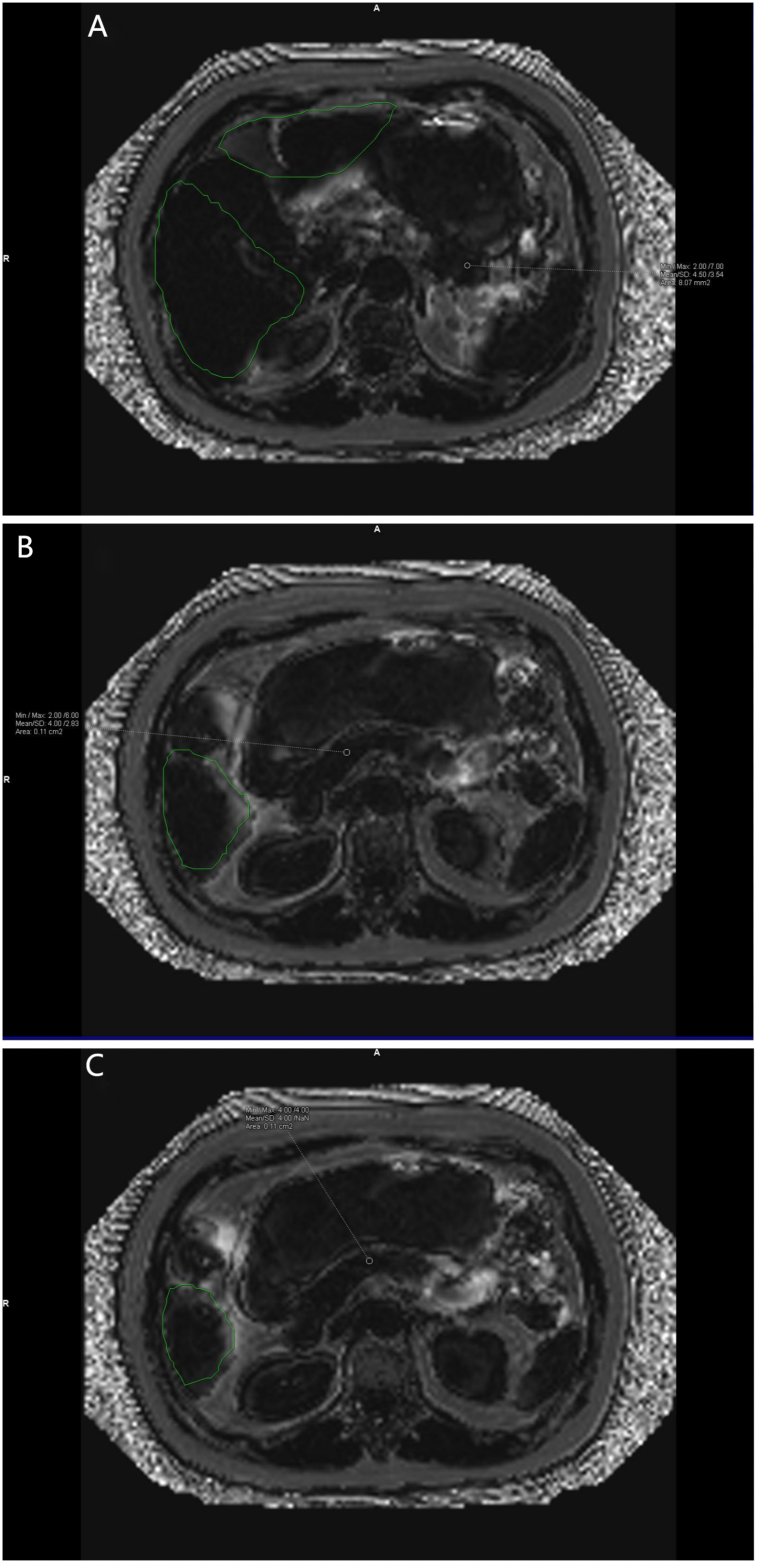
**(A–C)** show the corresponding Goodness of fit plots for pancreatic fat fraction. Goodness of fi mean = (4.5%+4.0%+4.0%)/3 = 4.17% (keep two decimal places). The green area is the area automatically delineated by the software.

**Figure 5:**
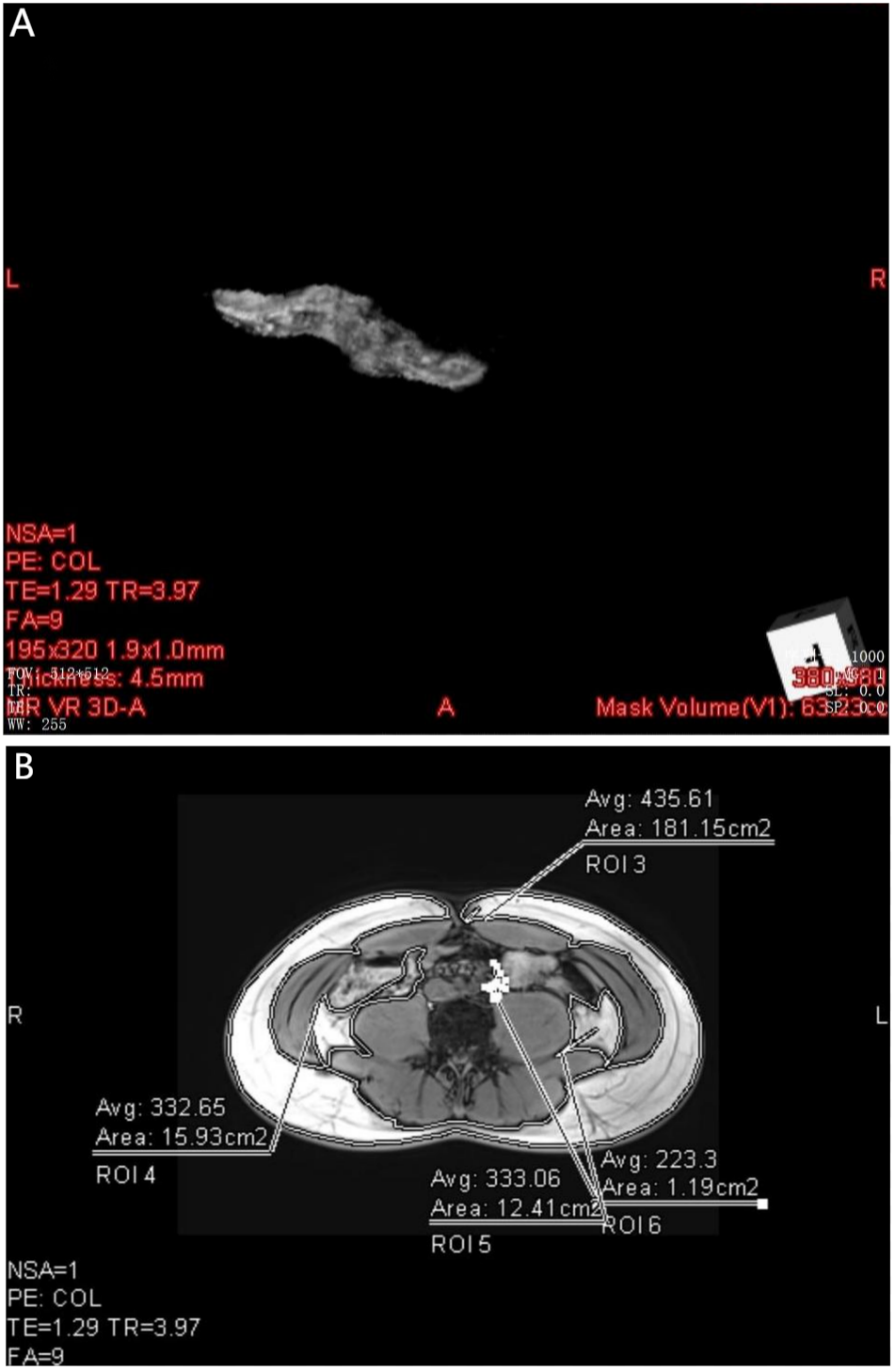
**(A)** shows the pancreatic Volume map of the volunteer the pancreas obtained by 3D standard processing, and the lower right corner of the figure shows the pancreatic volume Mask Volume(V1):63.23 cc. **(B)** shows the subcutaneous fat area(ROI 3 = 181.15cm^2^) and abdominal visceral fat area(ROI 4 = 15.93cm^2^, ROI 5 = 12.41cm^2^, ROI 6 = 1.91cm^2^) of volunteers after processing with Viewer.

Data measurements were performed by two radiologists who were familiar with image post-processing and had more than 5 years of experience in abdominal diagnosis. Measurement process: The Region of interest (ROI) was delineated independently on the fat content (FF) diagram of qDixon-WIP sequence, and the fat fraction was directly measured (fat fraction =10%× the mean measured by software). For liver, the intrahepatic sink area was avoided as far as possible. Four ROIs (liver S2/3, S4, S5/8, S6/7) were selected (**Figures 1A, B**) to measure liver fat fraction, each ROI was about 0.4 ~ 0.6cm2, and the corresponding Goodness of fit was measured (**Figures 2A, B**). Average values were taken (<5% indicates good accuracy). For the pancreas, three ROIs (head, body and tail of the pancreas) were selected (**Figures 3A–C**) to measure pancreatic fat fraction, each ROI was about 0.1-0.2 cm^2^, and Goodness of fit was also measured (**Figures 4A–C**), and average values were taken. The images were uploaded to Ziostation workstation (Ziostation2 Version 2.4.0.2), and the “3D standard and Viewer” functions in the workstation were used for image processing: The whole the pancreas was manually delineated, and the pancreatic volume was automatically calculated by the software (**Figure 5A**), and visceral fat area (VA) and subcutaneous fat area (SA) were measured in the experimental and control groups via the umbilical plane (**Figure 5B**). For pancreatic volume, in order to exclude the influence of height, weight and other factors among individuals, pancreatic volume to body surface area (PVI) was obtained by conversion (male: body surface area [m^2^] = 0.0057 × height [cm] + 0.0121 × weight [kg] + 0.0882; Female: body surface area [m^2^] = 0.0073 × height [cm] + 0.0127 × weight [kg] - 0.2106; Pancreatic volume per unit body surface area: PVI [cm^3^/m^2^]= pancreatic volume cm^3^/body surface area m^2^) (18). All measurement data were taken from the mean values measured by two doctors. The patient’s clinical data was queried through HIS system of our institution; Height and weight were measured on the day of MRI scan.

### 2.4 Statistical methods

SPSS22.0 software was used for statistical analysis. Kolmogorov-Smirnov(K) method was used to test the normal distribution of the data. Measurement data with normal distribution were represented as mean ± standard deviation (M). Measurement data with non-normal distribution were expressed as median, and quartile. Pearson chi-square test was used to compare the differences in gender composition. The independent sample t test (normal distribution) or Mann-Whitney U test (non-normal distribution) was used to compare the Pancreatic fat fraction (PFF), SA, VA and PVI between the experimental group and the control group. Pearson (normal distribution) or Spearman (non-normal distribution) correlation analysis was used to evaluate the correlation between the measured PFF and Hepatic fat component (HFF), PVI, SA, VA and clinical indicators in T2DM patients. The threshold for significance was set at 0.05.

## 3 Results

### 3.1 Consistency test for determination of pancreatic fat

The PFF, HFF, SA, VA and PVI of the experimental group and the control group were measured by two doctors (A and B) at different times. The Intraclass correlation coefficient (ICC) consistency test showed that the measured results were consistent between the groups (**Table 1**). It can be considered that the data measured by different doctors were highly consistent with intra-observer and inter-observer.

**Table 1:** Consistency test of PVI, subcutaneous fat area, abdominal fat area, PFF% and HFF% measured values between the experimental group and the control group by two doctors.

Group	Measurement index	Doctor A1	Doctor A2	Doctor B	Consistency coefficient (and 95% credibility Interval)	
					A1 and A2 A1 and B	
experimental	PVI (cm^3^/m^2^)	31.55 ± 1.79	31.58 ± 1.76	31.29 ± 1.73	0.993 (0.987~0.996)	0.917 (0.845~0.956)
	SA (cm^2^)	126.67 ± 44.70	126.65 ± 43.71	133.10 ± 45.05	1.000 (1.000~1.000)	0.957 (0.916~0.978)
	VA (cm^2^)	82.93 ± 26.48	82.94 ± 26.52	84.93 ± 22.59	1.000 (1.000~1.000)	0.925 (0.889~0.963)
	PFF (%)	3.70 (2.70∼5.40)	3.70 (2.50∼5.50)	3.08 (2.34∼5.90)	0.991 (0.984~0.995)	0.936 (0.893~0.971)
	HFF (%)	6.03 (3.975∼8.15)	6.00 (4.00∼8.50)	5.36 (3.48∼8.42)	0.994 (0.990~0.997)	0.949 (0.913~0.972)
control	PVI (cm^3^/m^2^)	33.68 ± 1.76	33.67 ± 1.76	34.48 ± 2.49	0.998 (0.996~0.999)	0.923 (0.861~0.958)
	SA (cm^2^)	108.56 ± 36.40	108.54 ± 36.41	107.95 ± 46.24	1.000 (1.000~1.000)	0.944 (0.891~0.975)
	VA (cm^2^)	37.31 ± 10.77	37.33 ± 10.76	38.52 ± 12.24	1.000 (1.000~1.000)	0.910 (0.829~0.945)
	PFF (%)	1.76 ± 0.66	1.79 ± 0.63	1.67 ± 0.69	0.992 (0.986~0.996)	0.950 (0.923~0.987)
	HFF (%)	3.37 ± 1.47	3.41 ± 1.49	3.13 ± 1782	0.999 (0.998~0.999)	0.933 (0.901~0.970)

PVI, ratio of pancreatic volume to body surface area; SA, subcutaneous fat area; VA, visceral fat area; PFF, pancreatic fat fraction; HFF, hepatic fat fraction.

### 3.2 Clinical parameter processing and normality test of measurement data

The normality test showed that BMI, PVI, SA, VA, TC, TG, HDL-c in the experimental group and BMI, PVI, SA, VA, PFF and HFF in the normal control group were all normal distribution (*P*>0.05). In the experimental group, PFF, HFF, FPC and LDL-c showed non-normal distribution (*P*<0.05) (**Table S1**).

### 3.3 Comparison and analysis results of related fat mass and parameters between T2DM patients and control group

There was no significant difference in age and gender distribution between the experimental group and the normal control group (*P*>0.05). The other indicators were BMI, PVI, SA, VA, PFF and HFF. There was no significant difference in BMI between the experimental group and the control group (*P*>0.05). There were statistical differences in PVI, SA, VA, PFF, and HFF between the two groups (*P*<0.05) (**Table 2**). PVI of T2DM patients was lower than that of control group, while SA, VA, PFF and HFF were higher than those of control group.

**Table 2:** Age, PFF, HFF, BMI, PVI and other indicators of T2DM patients and control group.

Evaluating indicator	T2DM	control group	T/X^2^	P
BMI(kg/m^2^)	23.04 ± 2.826	23.68 ± 2.327	-1.206	0.231
PVI(cm^3^/m^2^)	31.27 ± 1.761	34.01 ± 2.487	-6.220	<0.001
SA(cm^2^)	133.07 ± 44.91	108.44 ± 18.39	3.377	0.001
VA(cm^2^)	84.72 ± 22.30	37.91 ± 12.057	12.307	<0.001
PFF(%)	3.10(2.31∼5.84)	1.73 ± 0.697	-6.209	<0.001
HFF(%)	5.50(3.48∼8.42)	3.24 ± 1.617	-4.682	0.016
Age(year)	51.32 ± 10.604	51.28 ± 8.907	-0.024	0.981
gender	F=17,M=30	F=19,M=29	0.118	0.883
n	47	48	–	–

BMI, body mass index; PVI, patio of pancreatic volume to body surface area; SA, subcutaneous fat area; VA, visceral fat area; PFF, pancreatic fat fraction; HFF, hepatic fat fraction; n, example number.

### 3.4 Correlation analysis between fat-related measurements and clinical indicators in T2DM patients

**Figure 6:**
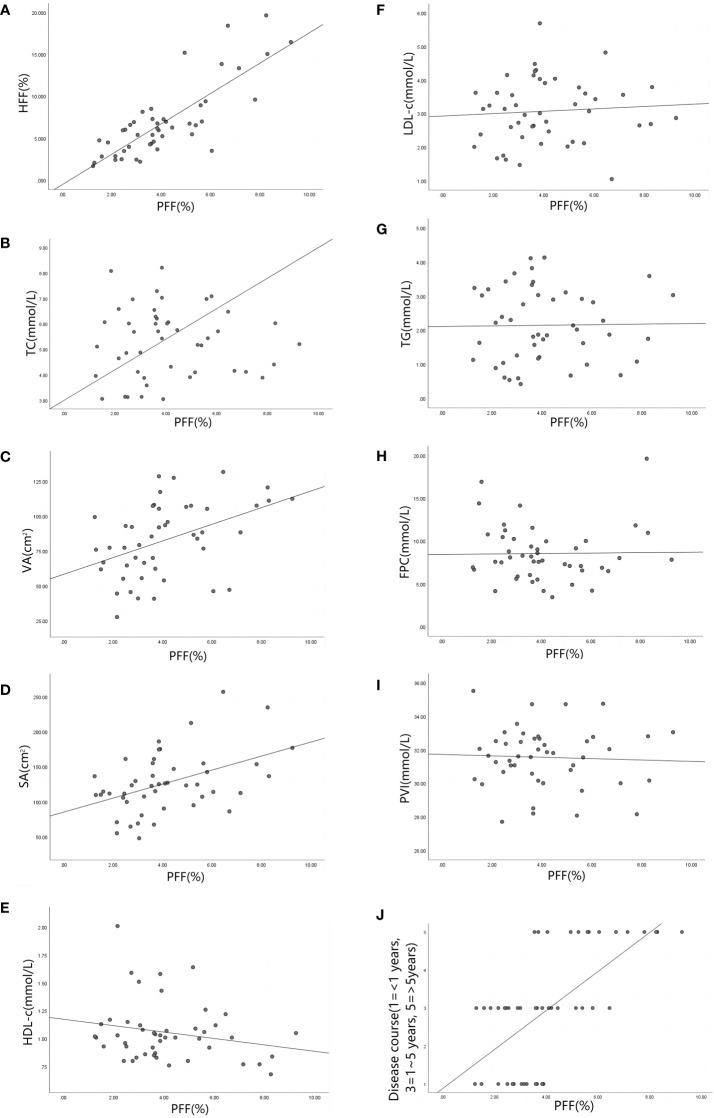
**(A–J)** are scatter plots of PFF and HFF(*r*=0.964, *P*<0.001), TC(*r*=0.236, *P*=0.437),VA(*r*=0.591, *P*<0.001), SA (*r*=0.321, *P*=0.033), HDL-c(*r*=-0.168, *P*=0.276), LDL-c(*r*=-0.002, *P*= 0.987),TG(*r*=0.676, *P*<0.001), FPC(*r*=0.385, *P*=0.194), PVI(*r*=-0.163, *P*=0.292) and Disease course(*r*=0.615, *P*<0.001) respectively. Note: pancreatic fat fraction (PFF), hepatic fat fraction (HFF), total cholesterol (TC), visceral fat area (VA), subcutaneous fat area (SA), high density lipoprotein (HDL-c), low density lipoprotein (LDL-c), triglyceride (TG), fasting blood glucose (FPC), patio of pancreatic volume to body surface area (PVI).

PFF was positively correlated with HFF in the experimental group (*r*=0.964, *P*<0.001). It was moderately positively correlated with TG, VA and Disease course (*r*=0.676, 0.591, 0.615, *P*<0.001), and weakly positively correlated with SA (*r*=0.321, *P*=0.033). There was no significant correlation with FPC, TC, PVI, HDL-c, LDL-c (*r*=0.385, 0.236, -0.163, -0.168, -0.002; P=0.194, 0.437, 0.292, 0.276, 0.987)(**Table 3** and **Figure 6**). The non-standardized linear regression equation constructed with PFF as the dependent variable and the other indicators as the independent variables is: PFF=10.287+0.284HFF-0.255PVI-0.329TG+0.758Disease course(According to the inspection level of 0.05, only HFF, PVI, TG and Disease course were included in the regression equation) (**Table 4**). The standardization coefficients of HFF, PVI, TG and Disease course are 0.637, -0.233, -0.18 and 0.303 (**Table 4**).

**Table 3:** PFF, HFF, abdominal wall, abdominal fat area and related clinical parameters in T2DM patients.

Clinical data	T2DM
FPC (mmol/L)	7.73 (5.61∼9.83)
TC (mmol/L)	5.02 ± 1.20
TG (mmol/L)	1.83 ± 0.958
LDL-c (mmol/L)	2.86 (2.34∼4.02)
HDL-c (mmol/L)	1.10 ± 0.296
PVI (cm^3^/m^2^)	31.27 ± 1.76
SA (cm^2^)	133.07 ± 44.91
VA (cm^2^)	84.72 ± 22.30
PFF (%)	3.10 (2.31∼5.84)
HFF (%)	5.50 (3.48∼8.42)
Age (year)	51.32 ± 10.604
gender	F=17,M=30
n	47

FPC, fasting blood glucose; TC, total cholesterol; TG, triglyceride; LDL-c, low density lipoprotein; HDL-c, high density lipoprotein; PVI, patio of pancreatic volume to body surface area; SA, subcutaneous fat area; VA, visceral fat area; PFF, pancreatic fat fraction; HFF, hepatic fat fraction.

**Table 4:** Linear regression relationship between PFF and different indicators in T2DM patients.

Index of correlation	Non-standardized coefficients	Standardization coefficient	t value	P value
Constant	10.287	-	2.122	0.041
AGE (years)	-0.036	-0.181	-1.712	0.096
BMI (kg/m^2^)	-0.05	-0.072	-0.917	0.366
HFF (%)	0.284	0.637	6.194	<0.001
PVI (cm^3^/m^2^)	-0.255	-0.233	-2.22	0.033
SA (cm^2^)	0.003	0.068	0.468	0.643
VA (cm^2^)	0.006	0.084	0.616	0.542
FPC (mmol/L)	-0.033	-0.056	-0.617	0.541
HDL-c (mmol/L)	0.709	0.096	0.956	0.346
LDL-c (mmol/L)	0.207	0.101	0.998	0.325
TC (mmol/L)	0.012	0.009	0.079	0.938
TG (mmol/L)	-0.329	-0.18	-2.074	0.046
Disease course (<1 years, 1~5 years, >5years)	0.758	0.303	3.117	0.004

BMI, body mass index; HFF, hepatic fat fraction; PVI, patio of pancreatic volume to body surface area; SA, subcutaneous fat area; VA, visceral fat area; FPC, fasting blood glucose; HDL-c, high density lipoprotein; LDL-c, low density lipoprotein; TC, total cholesterol; TG, triglyceride.

### 3.5 Comparison and analysis of PFF values between experimental group and control group in patients with different course of disease

The measurement results of PFF values in the control group and the experimental group with different course of disease are shown in **Table 5**. The results of comparison between groups are shown in **Table S2**. The PFF of the control group and the experimental group were statistically different (*P*<0.05), and the pancreatic fat content of the control group was lower than that of the experimental group. There was no statistically significant difference in PFF between patients with less than one year of disease course and those with one to five years of disease course in the experimental group (*P*>0.05), which could not indicate that the PFF of patients with one to five years of disease course was higher than that of patients with one year of disease course. The PFF of patients with less than 1 year and 1 to 5 years of disease course was statistically different from that of patients with more than 5 years of disease course (*P*<0.05), which could be considered that the PFF of patients with less than 1 year and 1 to 5 years of disease course was less than that of patients with more than 5 years of disease course.

**Table 5:** Kolmogorov-Smirnov(K) test of PFF values of T2DM patients and control volunteers with different disease stages.

Group	PFF (%)	Statistical variables	sample capacity	P value
control group	1.73 ± 0.697	0.098	48	0.200
disease course ≤1 year	2.28 (1.99∼3.45)	0.289	14	0.002
disease course of 1-5 years	2.75 (2.35∼4.40)	0.241	19	0.005
disease course > 5 years	6.46 ± 1.914	0.142	14	0.200

PFF, pancreatic fat fraction.

## 4 Discussion

For ectopic fat accumulation in T2DM, ectopic lipid deposition can promote its development and plays an important role in its progression (19, 20). Studies have reported that pancreatic fatty infiltration is associated with insulin resistance, and the incidence of diabetes in people with pancreatic fatty infiltration is significantly higher than the other people (21, 22). At present, MRI-based fat quantification technology can identify small changes in fat content, quantify fat and monitor steatosis, making it play an increasingly important role in the assessment of pancreatic fat content (10).

In this study, 3.0T MRI qDIXon-WIP sequence was used to quantify pancreatic fat, which improved the solution to the problem of inverse calculation of water image and fat image in qDixon image. Under the condition of good consistency of gender, age and BMI matching between the experimental group and the control group, the HFF, PFF and intraperitoneal and external fat contents of the experimental group were higher than those of the control group, which reflects that there is a certain connection between abnormal fat metabolism and ectopic fat deposition. The accumulation of lipids in the the pancreas can lead to the blockage of signaling pathways and insulin resistance, thus leading to the release of inflammatory adipokines, and ultimately aggravating the deposition of fat in the abdominal organs (17). However, abnormal glucose metabolism (decreased insulin secretion or insulin resistance) will lead to weakened liver cells’ ability to metabolize fat, resulting in increased ectopic fat deposition (17, 20–22).

In this study, the PFF value of T2DM group was almost 2 times that of the normal control group (**Table 2**), which is similar to the study conducted by Tushuizen et al (23). However, in this study, the data in T2DM group conformed to the normal distribution and the patient sample size was sufficient. In the experimental group, HFF and PFF of patients showed a strong positive correlation, suggesting that liver fat deposition was closely related to pancreatic fat deposition, which was similar to the research results of van Geenen (24). Some of the differences in results may be related to assessment methods (ultrasound, CT, magnetic resonance), individual differences (psychological factors, diet, exercise, BMI, etc.), measurement methods (delineation of areas of interest, uneven distribution of fat deposits in the the pancreas) and other factors. T2DM patients have abnormal metabolism, which will cause the increase of TG. When the TG in the body is supersaturation in adipose tissue, lipids will be accumulated in non-fatty organs, such as the pancreas, etc., and pancreatic fat infiltration will promote the progression of T2DM and the increase of TG (5). In the experimental group, the moderate positive correlation between PFF and TG indicates that they have a close relationship. The study of Hu and Yamazaki showed that abdominal fat accumulation and abdominal fat deposition were related to diabetes and other risk factors (25, 26). The study of Yu, Van and Anderson showed that SA and VA in T2DM patients were also related to T2DM: intra-abdominal fat decreased the inhibitory effect of insulin on lipolysis by increasing gluconogenesis and insulin sensitivity (27, 28). In this study, PFF was moderately positively correlated with abdominal fat and weakly positively correlated with subcutaneous fat area, which also reflected that intra-abdominal and extra-abdominal fat deposition were related factors for pancreatic fat infiltration. Some studies also pointed out that there was a significant correlation between abdominal fat distribution and older patients (29), and the different course of T2DM patients led to certain differences in results. In addition, this study also compared the correlation between PFF and FPC, PVI, HDL-c, TC and LDL-c, and the results indicated that there was no significant correlation. The constructed linear regression equation points out that among the relevant indicators in this study, HFF, PVI, TG and Disease course have greater contribution to PFF, that is, these four factors are closely related to PFF.

The patients in the experimental group were divided into three groups according to the course of disease: course of disease ≤1 year, 1 year < course of disease < 5 years, and course of disease ≥5 years. The results suggest that pancreatic fat accumulation is higher in patients with long course of T2DM than in those with short course of T2DM. According to the linear regression analysis, the standardized regression coefficient for Disease course was 0.303, which points out the degree of fat accumulation is higher in those with long-standing diabetes. And as mentioned above, insulin resistance causes ectopic fat deposition, and pancreatic fat also accumulates in the progression of T2DM. The results of this study may partly explain that pancreatic fat infiltration is a gradual accumulation process in patients with long disease course, but the degree of fat accumulation is slower in patients with short disease course.

Limitations of this study: (1) Due to the small sample size, further sample expansion is needed to improve the reliability of the experimental results. (2) Due to the age distribution characteristics of the diabetic population, the age of T2DM patients included in this study ranged from 32 to 71 years old, and the corresponding age of normal control population was matched, and the data obtained had certain bias. (3) In this study, T2DM patients were randomly sampled, and subgroup analysis of patients with different clinical interventions was not performed. The degree of pancreatic fat infiltration is likely to be different in patients with different interventions. This study can further focus on the relationship between pancreatic fat deposition and T2DM intervention.

In this study, the qDixon-WIP sequence was used to conduct clinical experiments. The results showed that: (1) PVI decreased, while SA, VA, PFF and HFF increased in T2DM patients. (2) PFF was positively correlated with HFF, TG, abdominal fat area and subcutaneous fat area in T2DM patients. (3) The degree of pancreatic fat accumulation in patients with long course of disease was higher than that in patients with short course of disease. This sequence can be used in clinical research to quantitatively measure pancreatic fat content with good repeatability, which can provide reference for clinical assessment of pancreatic fat to achieve real-time monitoring of the occurrence and progression of diseases.

## Data availability statement

The data analyzed in this study is subject to the following licenses/restrictions: The datasets generated during and/or analysed during the current study are not publicly available, but are available from the corresponding author on reasonable request. Requests to access these datasets should be directed to LiLing Long, cjr.longliling@vip.163.com.

## Ethics statement

The studies involving human participants were reviewed and approved by Ethics Committee of the First Affiliated Hospital of Guangxi Medical University (NO.2022-E460-01). Written informed consent for participation was not required for this study in accordance with the national legislation and the institutional requirements.

## Author contributions

Material preparation and data collection were performed by JY, FX, TL, SL and QF. Data analysis were performed by JY, FX. The first draft of the manuscript was written by JY, FX and BL and all authors commented on previous versions of the manuscript. All authors contributed to the article and approved the submitted version. LL contributed to the study conception and design.

## Supplementary material

Supplementary Table 1 Kolmogorov-Smirnov (K) test of normal distribution of experimental data.

Supplementary Table 2 Test results of PFF difference between T2DM patients and control group volunteers with different disease course.
