# Supplementary material for: Quantitative study of 3T MRI qDixon-WIP applied in pancreatic fat infiltration in patients with type 2 diabetes mellitus
Source: Front Endocrinol (Lausanne). 2023 Feb 17;14:1140111. doi: 10.3389/fendo.2023.1140111 (PMC9981945; doi:10.3389/fendo.2023.1140111)
Supplement: Supplementary Table 1 — Kolmogorov-Smirnov (K) test of normal distribution of experimental data. [file Table_1.docx]

**Table S1** Kolmogorov-Smirnov(K) test of normal distribution of experimental data

| Measurement index | Group | Sample capacity | Statistical variables | P value |
| --- | --- | --- | --- | --- |
| BMI | T2DM group | 47 | 0.079 | 0.200 |
|  | control group | 48 | 0.072 | 0.200 |
| PVI | T2DM group | 47 | 0.095 | 0.200 |
|  | control group | 48 | 0.063 | 0.200 |
| SA | T2DM group | 47 | 0.093 | 0.200 |
|  | control group | 48 | 0.126 | 0.066 |
| VA | T2DM group | 47 | 0.100 | 0.200 |
|  | control group | 48 | 0.121 | 0.088 |
| PFF | T2DM group | 47 | 0.210 | 0.000 |
|  | control group | 48 | 0.098 | 0.200 |
| HFF | T2DM group | 47 | 0.32 | 0.000 |
|  | control group | 48 | 0.114 | 0.173 |
| Age | T2DM group | 47 | 0.151 | 0.200 |
|  | control group | 48 | 0.116 | 0.164 |
| FPC | T2DM group | 47 | 0.149 | 0.016 |
| LDL-c | T2DM group | 47 | 0.135 | 0.044 |
| HDL-c | T2DM group | 47 | 0.114 | 0.179 |
| TG | T2DM group | 47 | 0.102 | 0.200 |
| TC | T2DM group | 47 | 0.092 | 0.200 |

Note: body mass index (BMI), patio of pancreatic volume to body surface area (PVI), subcutaneous fat area (SA), visceral fat area (VA), pancreatic fat fraction (PFF), hepatic fat fraction (HFF) , fasting blood glucose (FPC), low density lipoprotein (LDL-c), high density lipoprotein (HDL-c), triglyceride (TG), total cholesterol (TC).

**Table S2** Test results of PFF difference between T2DM patients and control group volunteers with different disease course

| Compare the group | N/N | Z | *P* |
| --- | --- | --- | --- |
| control group with disease course ≤1 year  control group with disease course of 1-5 years  control group with disease course > 5 years  disease course ≤1 year with disease course of 1-5 years  disease course ≤1 with disease course > 5 years  disease course of 1-5 years with disease course > 5 years | 48/14  48/19  48/14  14/19  14/14  19/14 | -3.219  -4.908  -5.429  -1.931  -3.998  -3.917 | 0.001  <0.001  <0.001  0.053  <0.001  <0.001 |

Note: pancreatic fat fraction (PF)
